# Physiology

**Published:** 1870-07

**Authors:** William T. Lusk

**Affiliations:** Professor of Physiology, Long Island College Hospital


### PHYSIOLOGY.

By WILLIAM T. LUSK, M.D., Professor of Physiology,
Long Island College Hospital.

1. *The Peptone Theory.*—Brücke, in a paper before the Academy of Sciences, in Vienna (*vide* Revue des Cours Scientifique, No. 50, 1869), expresses his dissent from the commonly-received opinion, that albuminoid substances necessarily undergo certain important changes before they become absorbed during digestion. The idea has its origin in the belief that diffusion and filtration take place in the peptones with much greater facility than in the albuminoids. Albuminoid substances pass with difficulty porous filters, because of their large sized molecules. But they are not all alike in this regard. The white of an egg passes through a cloth only as a consequence of strong pressure; whereas the speed with which the albumen of Wurtz passes through a filter proves that difficult filtration makes no part of the integral properties of natural albumen.

It is claimed that albuminoid substances must be transformed into peptones previous to absorption; and by peptones are understood modifications of albumen, which neither coagulate spontaneously, nor by heat, nor upon the addition of acids. This proposition is essentially erroneous. In animals killed during absorption, and kept twenty-four to forty-eight hours in a cold spot until the muscles were dead, Brücke found on opening the abdomen that the chyle was coagulated in the lacteals. This coagulation was most likely due to the presence of an acid, as the experiments were mostly made upon mammalia fed with milk, and the contents of the small intestine were likewise found in each case both acid and coagulated. The coagulation was not due to the admixture of lymph, as it extended to the ampullæ of Lieberkühn, from which upon sections the chyle could be pressed out in the form of filaments, visible under the microscope. Here the quantity of blood-plasma, escaped from the capillaries of the villi, must have been small, and it is known that albumen in blood-serum is only precipitated in minute quantities on the addition of a very dilute acid. The coagulation can hardly have been due to fibrin, for the chyle, taken from the lacteals of mesentery, and mingled with all the intestinal lymph, possesses but feeble coagulating powers. There is no doubt that the small intestine is capable of absorbing an albuminoid substance which does not possess the properties of the peptones. Aside from the albumen of the pancreatic juice, there is no difficulty in deriving from the nitrogenized alimenti a coagulable albuminoid matter capable of absorption. This we do by taking the liquid obtained by the digestion of meat before those modifications are produced, which result from the prolonged action of the gastric juice, neutralizing it until it is only feebly acid, when an albuminoid matter is precipitated, which again is soluble in an excess of alkali, and can afterward be reprecipitated from the alkaline fluid by rendering it once more feebly acid. This precipitate, which really forms when the bile flows into the duodenum, has furnished Meissner with his parapeptone theory. It may dissolve anew in the contents of the small intestine. The albuminoid substance thus precipitated and redissolved may become modified by the pancreatic juice; or it may be absorbed either wholly or in part before it has lost the property of separating upon the commencement of an acid reaction. If this reaction occurs after death, we would have at the same time coagulation of the chyle in the lacteals. But can albumen coagulable by heat be absorbed? The pancreatic juice contains an albuminoid body coagulable by heat, to which is attributed the *role* of emulsifying the fats. Fat is not only absorbed, but passes into the villi, carried along by the current of the liquid holding it in suspension. Now, we can hardly admit that passages large enough to permit the transit of fat-globules are too small to allow that of the molecules of the albumen, which has served for their emulsion, and if the passages allow the coagulable albumen of the pancreatic juice to traverse them, why not the albumen derived from food?

Bauer kept a dog fasting until the excretion of urea had become nearly constant. He then injected solutions of albumen into the large intestine, using sometimes peptones, sometimes the expressed acid muscular juice, sometimes albumen in a solution of salt, and finally albumen that had first been whipped, and then allowed to become fluid. In all of the three first instances, the large increase in the amount of urea excreted, proved that absorption had taken place.

Now, what is the state in which the albumen derived from food arrives in the intestines? Raw meat hashed, mixed with a good digestive fluid until the greater part has been dissolved, then filtered and neutralized until the precipitate, termed by

Meissner parapeptone, is formed, furnishes a fluid after further filtration, presenting a feebly acid reaction, which passes readily through a filter, and which coagulates with heat. This soluble albumen is found during artificial digestion for more than four hours, even when kept at the temperature of the body at which the peptonizing process is most active. Brücke says it is not the pepsine, but the acid, that takes from albumen its coagulabilty. To restore the latter, we need therefore to completely neutralize the fluid. Thus albumen remains soluble long enough to pass in that state into the intestine. A dog kept fasting for forty-eight hours, and then fed on raw meat, was killed two hours after the reception of food. The stomach contained a notable quantity of meat in fragments, and a liquid, which, after the addition of carbonate of soda, and the production of the precipitate of the neutralization, coagulated upon application of heat. The intestine contained a mucous liquid, rich in SOLUBLE albumen, and the microscope showed the presence of muscular fibres. Absorption was going on actively, and the lacteals were filled with chyle. Another modification of albumen was sometimes found in the stomach, distinguished from the foregoing, by the property of forming a precipitate of a gelatinous appearance, in adding to the neutral solution a weak solution of acetic acid. In this condition it coagulated by heat, a property not possessed when the solution presented a neutral reaction. This modification was probably in part due to the presence of an increased quantity of phosphatic salts.

After the ingestion of cooked albumen, however, the products of digestion, which have been operated upon by the gastric juice, furnish a precipitate when neutralized, but the filtered fluid no longer coagulates by heat. As man scarcely employs any thing but cooked food, it would seem that the soluble albumen of the system is regenerated either by the precipitable albumen, or by some foam of albumen so modified as not to coagulate by heat. But Kühne has found that the cooked fibrin of the blood is so modified by the pancreatic juice as to dissolve in a solution of common salt and form a liquid coagulable by heat; so it is probable that the pancreatic juice forms albumen coagulable by heat from the cooked albuminoid substances which pass undigested into the small intestine, and perhaps even from the precipitated albuminoid matters, which are found in the mass of the products of digestion after the action of the bile and the neutralization of the acid they contain. In the small intestine there is a notable quantity of soluble albumen, coagulable by heat, even when cooked meat has been used, which, however, proves nothing, as it may be derived from the pancreatic juice. Brücke obtained chyle from the lacteals by suction through a glass tube, and found that, when the contents were brought rapidly to the boiling-point in a tube placed in an oil-bath, they coagulated. But this may have been due, possibly, either to pancreatic juice, or to the lymph contained in the lacteals. This much, however, is certain, that there takes place an absorption of albuminoids which do not possess the properties of the peptones.

Brücke found it required fifteen hours to convert parapeptones into peptones at a temperature of 38° Celsius. Meissner says he never knew the transformation effected in so short a time. Mulder found that it required thirty-two hours, at a temperature of 40° Celsius, to completely change albuminoids into peptones. In the female, with the intestinal fistula observed by Busch, aliments began to pass out of the fistula at the end of from fifteen to thirty minutes, and digestion was completed, after a full repast, in three or four hours. In so short a time the conversion of albuminoids into peptones must have been relatively incomplete. It is well to notice, too, that after the patient had been strengthened by artficial alimentation through the lower part of the intestine, she was successfully nourished by the natural passages, notwithstanding the limited extent of the absorbent surface.

Admitting that the pancreatic juice continues the conversion into peptones, long before the albuminoid substances have had time to become materially modified, so soon in fact as the chyme reaches the small intestine, absorption begins; and if this portion of the intestine contains albuminoid substances in a state susceptible of absorption, they must necessarily be absorbed.

The question may be asked whether such albuminoid matters are really utilized by the economy—whether they do not disappear, while the albumen of the organism is regenerated by means of the peptones. When Busch first received his female patient, she was in a most deplorable condition. Believing that, so long as food was introduced by the natural passages only, she would not regain her strength, because of the shortness of the absorbent surface traversed, he introduced nitrogenized aliments by the fistula, so as to pass over the second portion of the intestinal canal. As this portion received neither gastric, nor pancreatic juice, were we to admit that all the albuminoid substances utilized after absorption consisted of peptones, we would have to presume a peptonizing activity on the part of the intestinal juice, in no wise warranted by our experience regarding it.

If albuminoid substances are exposed sufficiently long to the action of a digestive fluid, they become, according to Kühne, converted into leucine and tyrosine, yet no one would think of these substances as serving to regenerate the albuminoids of the economy. Yet in a series of metamorphoses why reject the first and last term, and fix upon the mean term, *viz.*, the peptones, as the sole regenerators. Simply, because they were thought to rank highest in the scale of the substances allied to albuminoid matters, which were capable of absorption; but, now that we recognize that other albuminoid substances, less modified in character, are capable of absorption, we must give to these the precedence over the peptones. There is no longer any reason for regarding the regeneration of the albuminoids as taking place at the expense of products of decomposition.

The question for us to solve is simply this. A series of albuminoid bodies and their products are absorbed during digestion. Which of these substances serve specially to regenerate the blood, the muscles, and the nerves? The limit ought to be fixed, beyond which the albuminoid substances cannot further be modified in the intestinal canal, and still preserve the property of regenerating the albuminoid substances which fulfil the *role* of functioning constituent elements of the organism. The solution is attended with labor and difficulty perhaps, but seems by no means impossible, through the agency of direct experiment upon animals provided with intestinal fistulæ.—*New York Medical Journal.*

